# Linarin alleviates colonic barrier dysfunction induced by enterotoxic *Escherichia coli* in weaned piglets by regulating the gut microbiota and metabolic pathways

**DOI:** 10.3389/fimmu.2025.1631991

**Published:** 2025-10-23

**Authors:** Qianqian Zhang, Xiaodan Liu, Chaofan Sun, Mingyang Wang, Xu Ji, Shenghe Li, Erhui Jin, Feng Zhang

**Affiliations:** ^1^ College of Animal Science, Anhui Science and Technology University, Chuzhou, China; ^2^ Anhui Province Key Laboratory of Livestock and Poultry Product Safety Engineering, Institute of Animal Science and Veterinary Medicine, Anhui Academy of Agricultural Sciences, Hefei, China; ^3^ Anhui Province Key Laboratory of Animal Nutrition Regulation and Health, Chuzhou, China

**Keywords:** enterotoxigenic *Escherichia coli*, linarin, weaned piglet, barrier function, metabonomic, correlation analysis

## Abstract

**Introduction:**

Enterotoxigenic *Escherichia coli* (ETEC) is a globally recognized gastrointestinal pathogen and a major cause of diarrhea in neonatal and post‐weaning animals, leading to significant economic losses in pig production. Premature weaning disrupts colonic morphology and barrier integrity, resulting in diarrhea, dehydration, growth retardation, and increased mortality. Linarin, a natural flavonoid derived from wild chrysanthemum, exhibits antioxidant, sedative, and anti‐osteoporotic properties, demonstrating potential as a therapeutic agent and functional food ingredient.

**Methods:**

24 healthy 21‐day‐old weaned piglets (Duroc × Landrace × Large Yorkshire) were randomly assigned to four groups fed a basal diet (BD) or linarin-supplemented diet (LN) with oral infusion of 10 mL nutrient broth (NB) or 10^⁹^ colony-forming units/mL ETEC. Following a 3‐day acclimation period, piglets were fed the corresponding diet for 21 days; infusion with ETEC or NB was performed for 3 days on days 8 and 18. Colonic morphology, diarrhea incidence, gene expression, short-chain fatty acids (SCFAs), microbiota composition, and metabolomic profiles were assessed.

**Results:**

Linarin supplementation significantly ameliorated colonic crypt hyperplasia, increased goblet cell numbers, and decreased diarrhea incidence following ETEC infusion. It downregulated pro‐apoptotic and pro‐inflammatory gene expression while upregulating barrier‐associated genes. Linarin also significantly increased the concentrations of short-chain fatty acids (acetic, propionic, valeric, and isovaleric acids) in the colon. Integrated analysis of 16S rRNA gene sequencing and non-targeted metabolomics revealed that linarin modulated the intestinal microbiota by altering the relative abundance of key bacterial taxa (*Pedosphaera, Fusicatenibacteria, Tyzerella, Sporobacteria, Limosilactobacillus, Senegalimassilia, Catenibacillus*, and *Bryobacteria*), and associated metabolic pathways, including purine and pyrimidine metabolism; steroid, porphyrin, and vitamin biosynthesis; various amino acid and nucleotide metabolic processes; unsaturated fatty acid biosynthesis; and the citric acid cycle.

**Discussion:**

These findings indicate that linarin restores colonic barrier function and intestinal microbiota homeostasis, enhancing resistance to ETEC infection along with the development and well-being of piglets after weaning. This study offers a new mechanistic understanding of how linarin confers protection against ETEC, which can promote its widespread application as a natural feed additive to replace antibiotics.

## Graphical Abstract

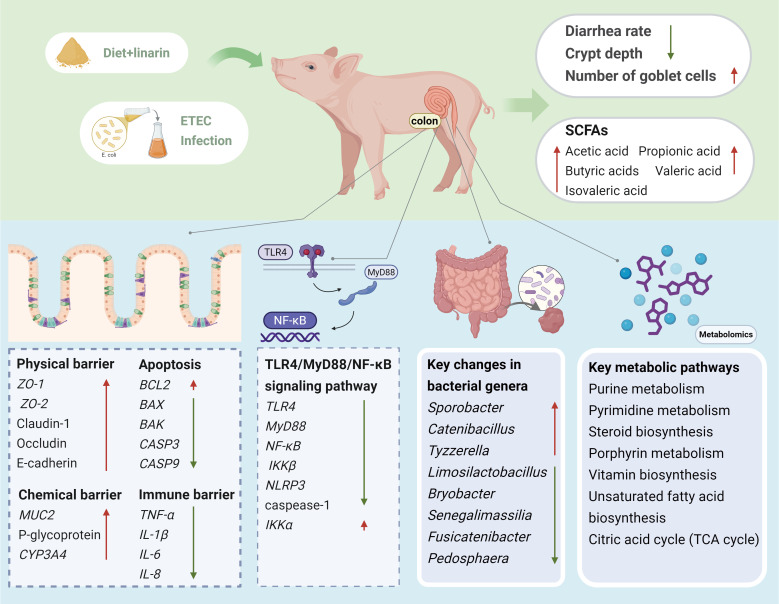

## 1 Introduction

The premature weaning of piglets often disrupts the colonic morphology and impairs intestinal barrier function, leading to diarrhea, dehydration, growth retardation, and increased mortality (1). In particular, post-weaning diarrhea (PWD) causes slow growth and a decreased feed conversion rate in piglets, resulting in serious economic losses and reduced production efficiency in the swine industry (1). Enterotoxigenic *Escherichia coli* (ETEC) is a major gastrointestinal pathogen that impairs intestinal function, which is a leading cause of diarrhea in neonatal and post-weaning animals. ETEC infection can lead to oxidative stress, disruption of intestinal barrier integrity (2), and cell death and tissue damage (3). ETEC adheres to intestinal epithelial cells using a colonization factor to colonize the small intestine of humans and animals, especially piglets and calves (4). As ETEC infection is one of the major causes of economic losses in pig production worldwide, safe and alternative treatment methods are needed.

ETEC K88 has F4 pili, facilitating binding to specific receptors on the surface of the pig intestine (5). The intestinal microbiota consists of a wide range of bacteria in the intestinal tract, mainly in the colon (6). The intestinal microbiota contribute to formation of the intestinal barrier; thus regulating the equilibrium of colonic microbial communities can help maintains the balance of nutrient metabolism, prevents disease, and achieve healthy growth (7). The health status of the gut determines the health status of the animal and is closely related to its production level and efficiency. Therefore, it is important to discover effective functional nutrients to mitigate intestinal damage in piglets.

Medicinal and dietary homology is a foundational concept in traditional Chinese medicine, which refers to the dual functionality of natural substances that serve both therapeutic and nutritional purposes (8). Linarin, a natural flavonoid abundant in wild chrysanthemums (9), has received increasing attention for its pharmacological activities. Linarin glycosides possess antioxidant properties, sleep-promoting and sedative effects, and anti-osteoporotic activity (10). One study showed that linarin had anti-inflammatory effects by inhibiting activation of the TXNIP/NLRP3 inflammasome, NF-κB pathway, and the release of various pro-inflammatory cytokines such as interleukin (IL)-1β, tumor necrosis factor (TNF)-α, and IL-6 (11). Another study showed that linarin reduced the expression level of Toll-like receptor 4 (TLR4) and its downstream factors MyD88, IRAK1, and TRAF6, thereby reversing the excessive phosphorylation of ERK, p38, and JNK to ultimately inhibit the inflammatory signaling cascade (12). Notably, linarin alleviated dextran sulfate sodium-induced colitis in mice (13). Our previous studies showed that ETEC infection disrupts the gut microbiota and activates TLR4/MyD88/NF-κB signaling, which causes gut inflammation in piglets (14). In addition, ETEC exposure may activate the Nrf2 antioxidant pathway as a compensatory response to infection-driven oxidative stress, potentially mitigating intestinal damage (14). To our knowledge, no study has directly assessed the effects of linarin on the intestinal barrier integrity of weaned piglets. To fill this gap, the aim of this study was to explore the potential of linarin to inhibit ETEC infection, consequently improving the intestinal health and breeding benefits of weaned piglets. Toward this end, we used a weaned piglet model infected with ETEC to investigate the effects of linarin on the diarrhea rate, histomorphology, barrier function, apoptosis, intestinal microbiota, and metabolic pathways, offering a potential alternative to conventional antibiotic-based treatments for ETEC-induced diarrhea.

## 2 Materials and methods

### 2.1 Animal ethics

The experimental animals (piglets) used in this study were privately owned. Animal protocols received approval from Experimental Animal Ethics Committee of Anhui Science and Technology University (approval no AHSTU2023006), and were strictly followed local regulations and relevant institutional norms. Animals participating in this study have obtained written informed consent from their owners.

### 2.2 Bacterial strains

Linarin (Chengdu Zhibiaohua Pure Biotechnology Co., Ltd., Chengdu, China; CAS No. 480-36-4, HPLC ≥ 90%). The *E. coli* F4 (K88 ac) strain was obtained from CVCC1500 (China Veterinary Culture Collection Center).

### 2.3 Animal experiment design

**Figure 1:**
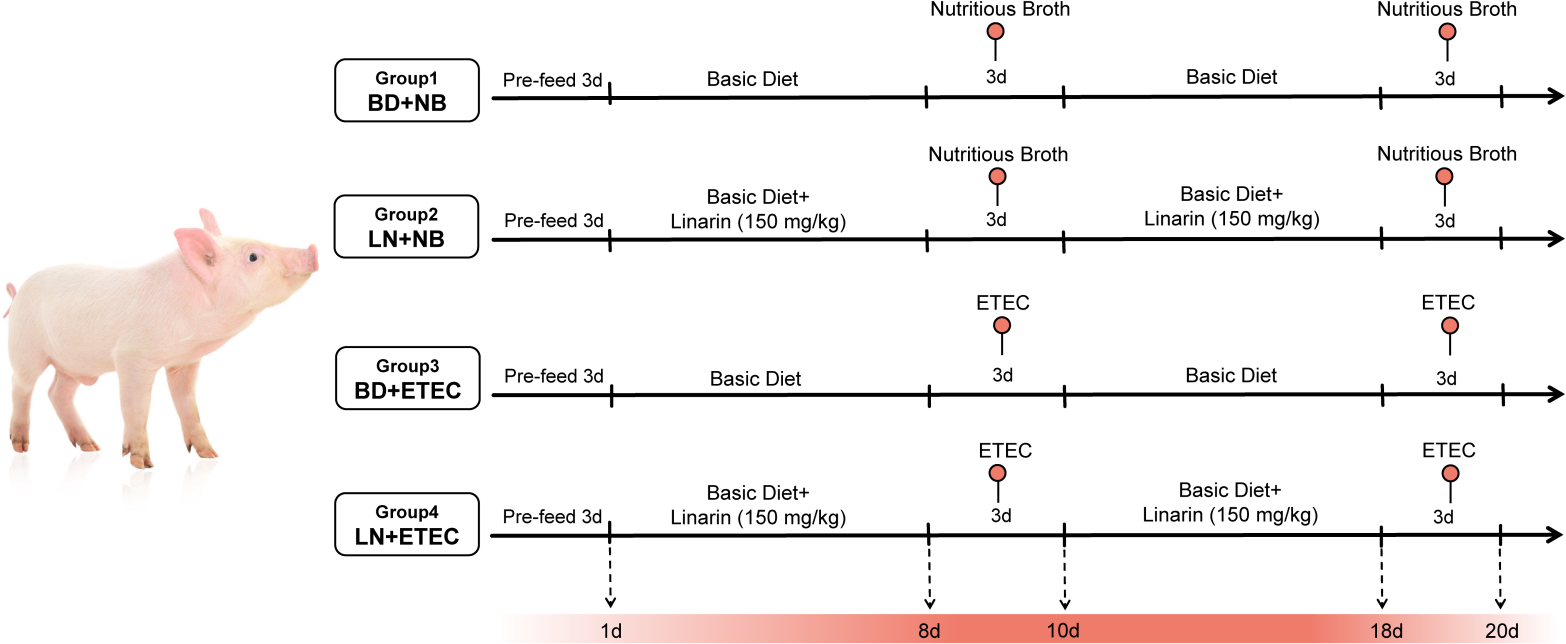
Design of animal experiments. BD, basal diet; LN, basal diet with linarin (150 mg/kg); NB, nutrient broth.

Twenty-four healthy (Duroc × Landrace × Large Yorkshire; average initial body weight: 6.45 ± 0.18 kg) weaned piglets at 21 days of age were selected and randomly divided into four treatment groups, each with 6 piglets (6 replicates, half male and half female), and reared in separate pens, while there was no significant difference in the initial body weight of the piglets among the treatment groups. The treatment groups were set up as follows: (i) Control group (BD+NB), basal diet with oral nutrient broth for piglets; (ii) linarin group (LN+NB), basal diet with 150 mg/kg linarin; (iii) ETEC group (BD+ETEC), basal diet with oral ETEC solution; (iv) linarin + ETEC group (LN+ETEC), basal diet with 150 mg/kg linarin, with oral ETEC solution. The experimental design included a 3-day acclimation phase preceding a 21-day trial period. During the experimental phase, piglets in the BD+ETEC and LN+ETEC groups received oral administration of 10 mL ETEC suspension (10^9^ CFU/mL) on days 8 and 18, while those in the BD+NB group were given 10 mL nutrient broth daily for three consecutive days (**Figure 1**). The linarin dosage was determined based on previous studies in mouse colitis models, with conversion to piglet-equivalent doses using surface area normalization (13, 15). The ETEC challenge dose and duration was established according to our previous research (16). Feed and water were available for piglets to consume freely during the test period. After the test ended, colon samples from the piglets were collected and maintained for later investigation. The experimental diet was provided in powdered form, diet composition and nutrients are detailed in **Supplementary Table S1**.

### 2.4 Diarrhea rate of piglets

Pre feeding period was 3 days, and the feeding trial was 21 days, the number of piglets with diarrhea was observed and the diarrhea score was recorded every day (0 = normal, hard feces; 1 = soft, probably mild diarrhea; 2 = apparently unformed, moderate diarrhea; 3 = feces very much watery and foamy, severe diarrhea), via the formula below. Refer to previous articles published in our laboratory (16),


$$\begin{array}{c} Diarrhea\;rate\;\left(\%\right)\;=\;\left(Number\;of\;piglets\;with\;diarrhea\;\times \;number\;of\;days\right)/\\ \left(Total\;piglets\;\times \;total\;trial\;days\right)\;\times \;100 \end{array}$$

### 2.5 Sample collection and processing

On the 25th day, piglets were treated humanely through the administration of sodium pentobarbital (40 mg per kilogram of body weight), after which dissection was performed. Blood was collected and allowed to clot at room temperature, then spun at high speed (3500 rpm) for 15 minutes. Centrifugation was carried out to yield serum, which was stored at -20°C. In addition, colonic tissue was collected from all piglets, and segments approximately 3 cm from the center of each colon were immediately isolated, rinsed with saline, and fixed in 4% paraformaldehyde solution for morphological examination and immunohistochemical analysis.

### 2.6 Morphological analysis of the colon

Colon specimens were fixed in 4% paraformaldehyde (pH 7.4) for 24 h, paraffin-embedded, and sectioned at 4 μm thickness. Sequential staining with hematoxylin & eosin (H&E) and Alcian blue periodic acid-Schiff staining (AB-PAS) was performed to evaluate crypt architecture and goblet cell density. For quantitative analysis, four representative fields per section meeting selection criteria (intact mucosal surface, vertically oriented crypts) were imaged at 200× magnification. Using ImageJ software (version 1.53; National Institutes of Health, Bethesda, MD, USA, crypt depth (measured from crypt base to luminal opening) and goblet cell counts (quantified as AB-PAS^+^ cells per crypt) were assessed in 30 intact crypts per sample.

### 2.7 Real-time PCR

Colon tissue RNA was extracted using the RNAprep Pure Tissue Kit (Tiangen Biotech, Beijing, China; Cat. No. DP431), followed by genomic DNA elimination, and 4 μL (1000 ng) of total RNA was reverse transcribed with HiScript III RT SuperMix (Vazyme Biotech, Nanjing, China; Cat. No. R323-01). QuantStudio 5 system (Applied Biosystems, USA) equipped with ChamQ SYBR qPCR Master Mix (Vazyme Biotech Co., Ltd., Nanjing, China; Cat. No. Q311-02) was used to conduct qPCR amplification. Primers were designed with reference to previously published studies from our research team (14, 16), utilizing NCBI Primer−BLAST, the designed primers were validated for specificity using BLAST. Standard-curve efficiency analysis was not performed in this study. Instead, amplification specificity and efficiency consistency were verified through melt-curve and Ct analyses. Each primer pair produced a single, well-defined melt-curve peak without nonspecific amplification or primer-dimer formation. The Ct values for individual genes remained within a narrow and consistent range across biological replicates, supporting the assumption of comparable amplification efficiency among reactions. Therefore, Relative mRNA expression was calculated using the 2^−ΔΔCt^ method, with the control group serving as the calibrator and glyceraldehyde-3-phosphate dehydrogenase (GAPDH) and β-actin as endogenous controls.

### 2.8 Determination of SCFAs

Colonic chyme samples were analyzed for short-chain fatty acid (SCFAs) encompassing acetate, propionate, butyrate, isovaleric, and valeric acids. Fresh samples were homogenized with ddH_2_O, centrifuged (10,000 rpm, 10 min), and supernatants were acidified with 25% metaphosphoric acid (4°C, 2 h), followed by centrifugation (20,000 × g, 10 min, 4°C). The 0.45 μm filtered supernatants were measured employing the Agilent 6890 Gas Chromatography system (manufactured by Agilent Technologies, United States), with concentrations expressed as mg/g.

### 2.9 Intestinal microbiota analysis

The intestinal microbiota was analyzed following the methods described in previous studies (17, 18). Microbial DNA was extracted from colonic samples using the E.Z.N.A.^®^ Stool DNA Kit (Omega Bio-tek, Norcross, GA, U.S.; Cat. No. D4015-02) according to manufacturer’s protocols. The V4-V5 regions of bacterial 16S rRNA genes were amplified using primers 341F (CCTACGGGNGGCWGCAG) and 806R (GGACTACHVGGGTATCTAAT), with unique 8-bp barcodes for each sample. Paired-end sequencing (2×250 bp) was conducted on an Illumina MiSeq platform. Raw sequencing data are available in the NCBI SRA database (BioProject: PRJNA1263251; BioSample: SAMN48516502; SRA: SRR33580208). Microbial diversity was evaluated using Chao, ACE, and Shannon indices.

### 2.10 LC-MS-based untargeted metabolomics

Metabolites were extracted from colon tissues, serum, microbial cultures using prechilled 80% methanol with 0.1% formic acid (19, 20). Tissue samples (100 mg) were homogenized in liquid nitrogen, while liquid samples (100 μL) and cell/bacterial pellets were mixed with methanol, vortexed, and centrifuged (15,000 rpm, 4°C) (21). Supernatants were diluted to 53% methanol, filtered, and analyzed via UHPLC-MS/MS (Vanquish system; Hypersil Gold C18 column) under a 17-min gradient (22). Mass spectrometry (Orbitrap Q Exactive™ HF) operated in positive/negative modes with a spray voltage of 3.2 kV and a capillary temperature of 320°C was employed, and a m/z 100–1500 scan range.

Raw data were processed using Compound Discoverer 3.1 for peak alignment (mass tolerance: 5 ppm; S/N ≥ 3), annotated via mzCloud, HMDB, and LIPID Maps databases. Significant metabolic markers (VIP: ≥ 1, *P-*value: < 0.05, at least a 2-fold increase or a 0.5-fold decrease) were identified using PCA/PLS-DA (metaX) and enriched in KEGG pathways (*P* < 0.05). 

### 2.11 Statistical analysis

Data were first tested for normality and transformed as needed. Two-way ANOVA was used to assess group differences. For non-normally distributed data (assessed by Shapiro-Wilk test, *P* < 0.05), including gut microbiota relative abundance, we applied the Kruskal-Wallis test. For the Pearson correlation analysis between microbial taxa and metabolites (or other variables), multiple testing correction was applied using the False Discovery Rate (FDR) method (Benjamini-Hochberg procedure), with significance thresholds set at *P* < 0.05 (statistically significant) and *P* < 0.01 (extremely statistically significant). Data analysis used SPSS 26.0, while GraphPad Prism 10.0 created visualizations.

## 3 Result

### 3.1 Effects of linarin on the ETEC-induced diarrhea rate

As shown in **Table 1**, piglets in the LN+ETEC group exhibited significantly lower diarrhea rates than those in the BD+ETEC group at 1–10 d, 11–20 d, and 1–20 d (*P* < 0.01), whereas no significant difference in diarrhea rate was observed between the LN+NB and BD+NB groups (*P* > 0.05).

**Table 1:** Effect of linarin on diarrhea rate in weaned piglets after ETEC infection.

Measure (%)	Experimental diet				SEM	*P*-value		
	BD+NB	LN+NB	BD+ETEC	LN+ETEC		Diet	ETEC	Interaction
1-10d	5.25^b^	3.70^b^	10.49^a^	4.94^b^	0.77	0.36	<0.01	0.10
11-20d	2.99^b^	3.09^b^	9.26^a^	5.56^b^	0.68	0.94	<0.01	0.05
1-20d	4.14^bc^	3.40^c^	9.88^a^	5.24^b^	0.59	0.39	<0.01	<0.01

^a,b,c^Values ​​with different superscripts in the row indicate significant differences (*P* < 0.05).

### 3.2 Effects of linarin and ETEC on colonic histomorphology and goblet cells

**Figure 2:**
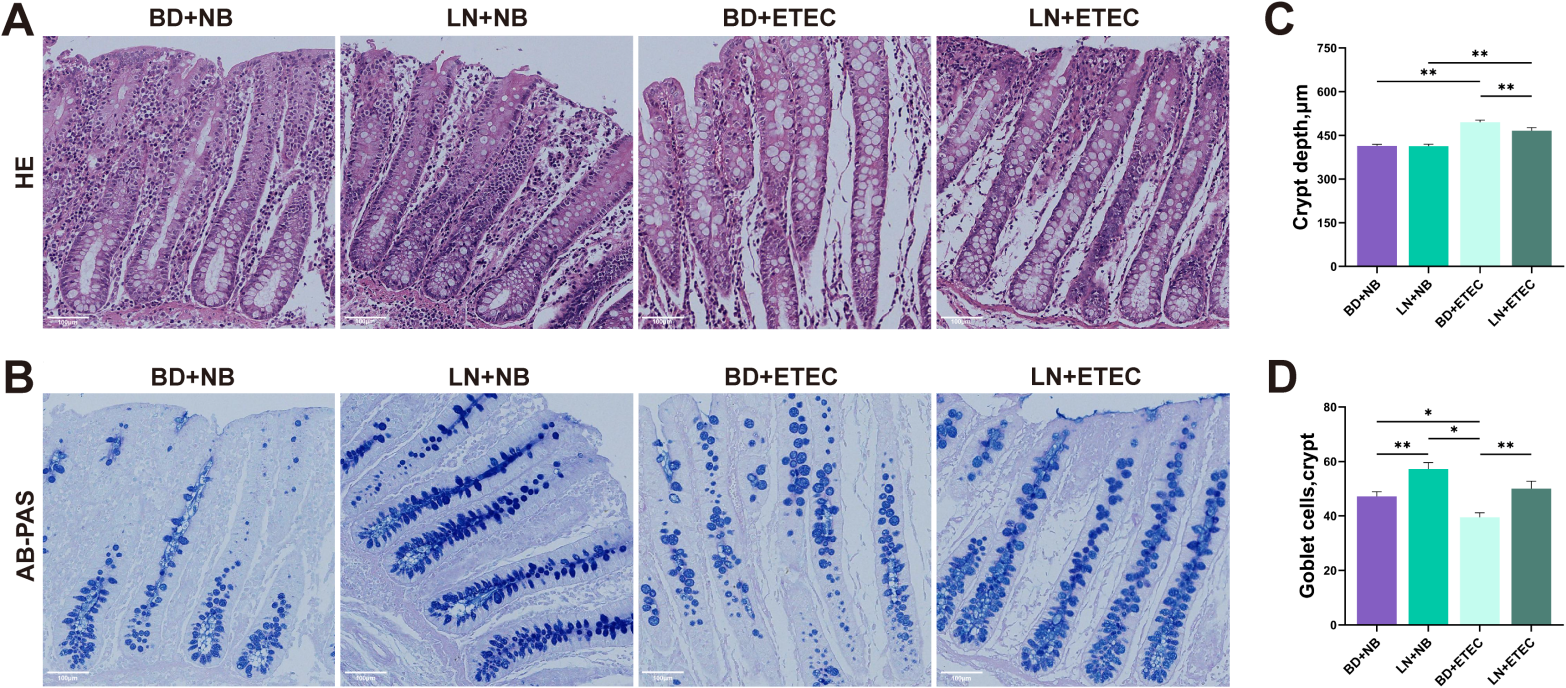
Effects of dietary linarin supplementation on colonic histomorphology and goblet cells of weaned piglets after ETEC infection. **(A)** The colon tissue was subjected to representative hematoxylin and eosin (HE) staining. **(C)** Crypt depth, 100 μm. **(D)** Goblet cell count. BD+NB, piglets received the basal diet + oral nutrient broth; LN+NB, piglets received the basal diet supplemented with 150 mg/kg linarin + oral nutrient broth; BD+ETEC, ETEC-challenged piglets fed the basal diet; LN+ETEC, piglets with ETEC infection fed the basal diet + 150 mg/kg linarin. Significance thresholds are set at *P* < 0.05 (statistically significant) and *P* < 0.01 (extremely statistically significant). *: *P* < 0.05 (statistically significant), **: *P* < 0.01 (highly statistically significant).

There was no significant difference in crypt depth between the BD+NB and LN+NB groups (*P* > 0.05) (**Figure 2C**). However, the crypt depth in the LN+ETEC group was decreased compared to that of the BD+ETEC group (*P* < 0.01) (**Figure 2C**). In addition, compared to the BD+NB group, goblet cells numbers in the LN+NB group were increased (*P* < 0.01) (**Figure 2D**). Similarly, goblet cell numbers in the BD+ETEC group were increased compared to those of the BD+ETEC and LN+ETEC groups (*P* < 0.01) (**Figure 2D**).

### 3.3 Effects of linarin and ETEC on the colonic physical barrier

**Figure 3:**
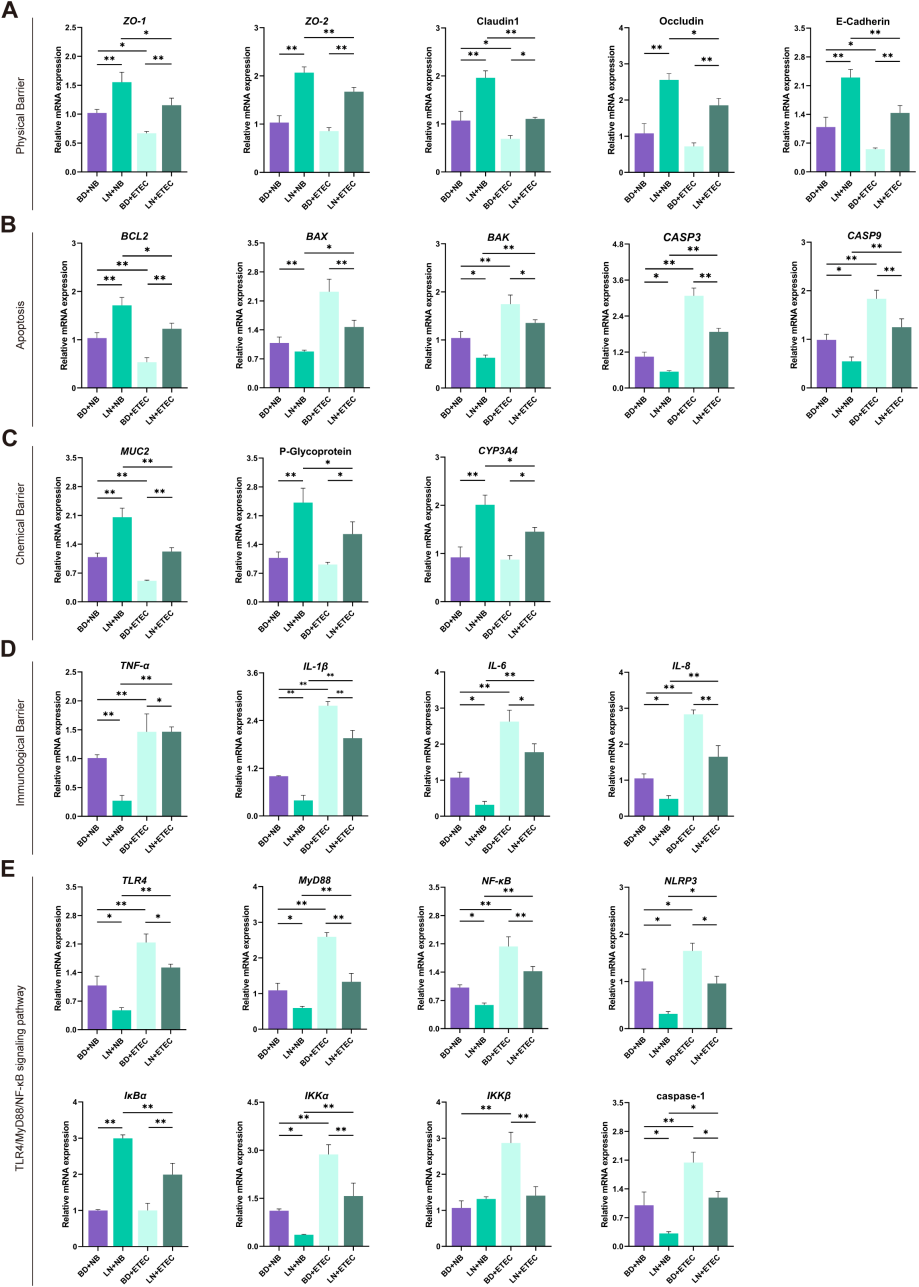
Effects of dietary linarin supplementation on colonic barrier integrity and inflammatory signaling in weaned piglets after ETEC challenge. The mRNA expresssion levels of **(A)** physical barrier-related genes (*ZO-1*, *ZO-2*, claudin-1, occludin, E-cadherin), **(B)** apoptosis regulators (*BCL2*, *BAX*, *CASP3*, *CASP9)*, **(C)** chemical barrier-related genes (*MUC2*, P-glycoprotein, *CYP3A4*), **(D)** immune barrier-related genes (*TNF-α*, *IL-1β*, *IL-6*, *IL-8*), and **(E)** TLR4/MyD88/NF-κB pathway genes (*TLR4*, *MyD88*, *NF-κB*, *NLRP3*, *IκBα*, *IKKα*, *IKKβ*, caspase-1). BD+NB, piglets received the basal diet + oral nutrient broth; LN+NB, piglets received the basal diet supplemented with 150 mg/kg linarin + oral nutrient broth; BD+ETEC, ETEC-challenged piglets fed the basal diet; LN+ETEC, piglets with ETEC infection fed the basal diet + 150 mg/kg linarin. Significance thresholds are set at *P* < 0.05 (statistically significant) and *P* < 0.01 (extremely statistically significant). *: *P* < 0.05 (statistically significant), **: *P* < 0.01 (highly statistically significant).

The mRNA expression levels of *ZO-1*, *ZO-2*, claudin-1, occludin, and E-cadherin were increased in the LN+NB group compared to those of the BD+NB group (*P* < 0.01). Similarly, compared to those of the BD+ETEC group, the mRNA expression levels of *ZO-1*, *ZO-2*, E-cadherin (*P* < 0.01), and occludin (*P* < 0.05) were increased in the LN+ETEC group (**Figure 3A**).

### 3.4 Effects of linarin and ETEC on apoptosis gene expression in colonic epithelial cells

Compared to that of the BD+NB group, the mRNA expression level of *BCL2* (*P* < 0.01) was increased, whereas the levels of *BAX* (*P* < 0.01), *BAK*, *CASP3*, and *CASP9* (*P* < 0.05) were significantly decreased in the LN+NB group. Similarly, compared to that of the BD+ETEC group, the mRNA expression level of *BCL2* was increased (*P* < 0.01), whereas the levels of *BAX*, *CASP3*, *CASP9* (all *P* < 0.01), and *BAK* (*P* < 0.05) were significantly decreased in the LN+ETEC group (**Figure 3B**).

### 3.5 Effects of linarin and ETEC on the colonic chemical barrier

The mRNA expression levels of *MUC2*, P-glycoprotein, and *CYP3A4* were significantly higher in the LN+NB group than in the BD+NB group (*P* < 0.01). The mRNA expression levels of *MUC2* (*P* < 0.01), P-glycoprotein, and *CYP3A4* (*P* < 0.05) were also significantly higher in the BD+ETEC group compared to those of the LN+ETEC group (**Figure 3C**).

### 3.6 Effects of linarin and ETEC on the colonic immune barrier

Compared with those of the BD+NB group, the mRNA expression levels of *TNF-α*, *IL-1β* (*P* < 0.01), *IL-6*, and *IL-8* (*P* < 0.05) were decreased in the LN+NB group. Similarly, compared to those of the BD+ETEC group, the LN+ETEC group had decreased levels of *IL-1β*, *TNF-α*, *IL-6*, and *IL-8* (*P* < 0.05 or *P* < 0.01) (**Figure 3D**).

### 3.7 Effects of linarin and ETEC on genes involved in the TLR4/MyD88/NF-κB signaling pathway

Compared with those of the BD+NB group, the mRNA expression levels of *TLR4*, *MyD88*, *NF-κB*, *NLRP3*, *IKKα*, and caspase-1 were significantly decreased (*P* < 0.05), whereas the level of *IκBα* was increased (*P* < 0.01) in the LN+NB group. Compared with those of the BD+ETEC group, the mRNA expression levels of *MyD88*, *NF-κB*, *IKKα*, *IKKβ* (*P* < 0.01), *TLR4*, *NLRP3*, and caspase-1 (*P* < 0.05) were decreased, whereas the level of *IκBα* was increased (*P* < 0.01) in the LN+ETEC group (**Figure 3E**).

### 3.8 Effects of linarin and ETEC on colonic short-chain fatty acids

As shown in **Table 2**, relative to the BD+NB group, the LN+NB group exhibited a highly significant (*P* < 0.01) increase in the levels of acetic, propionic, and butyric acids, along with a significant (*P* < 0.05) increase in the level of isovaleric acid. However, the concentration of valeric acid did not differ between the two groups. Compared with those of the BD+ETEC group, the LN+ETEC group showed increased levels of acetic, propionic, butyric, valeric (*P* < 0.01), and isovaleric (*P* < 0.05) acids.

**Table 2:** Effects of linarin on colonic short-chain fatty acid levels in piglets after ETEC infection.

Measure	Experimental diet				SEM	*P*-value		
	BD+NB	LN+NB	BD+ETEC	LN+ETEC		Diet	ETEC	Interaction
Acetate acid	50.41^b^	58.94^a^	43.05^c^	55.01^b^	1.29	<0.01	<0.01	0.05
Propionic acid	19.50^b^	22.50^a^	16.36^c^	19.78^b^	0.57	<0.01	<0.01	0.77
Butyric acid	5.59^b^	9.10^a^	4.38^c^	7.52^b^	0.40	<0.01	<0.01	0.56
Isovaleric acid	1.66^b^	2.16 ^a^	1.18^c^	1.42^b^	0.10	0.01	0.04	0.70
Valeric acid	1.23 ^b^	1.28^b^	1.16^b^	2.30^a^	0.12	0.8	<0.01	<0.01

^a,b,c^Values ​​with different superscripts in the row indicate significant differences (*P* < 0.05).

### 3.9 Effects of linarin and ETEC on the colonic biological barrier

**Figure 4:**
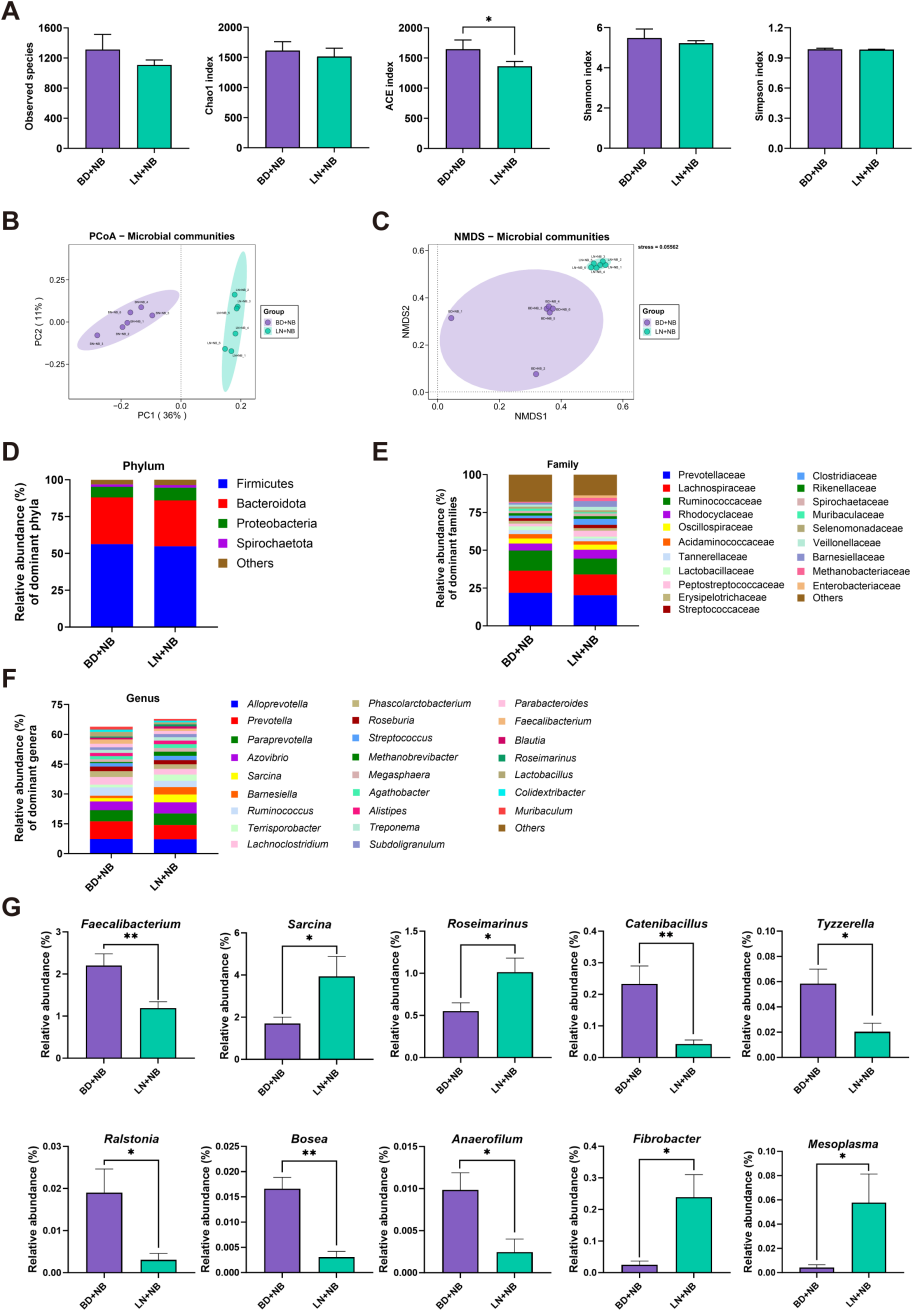
Effects of dietary linarin on weaned piglet microbiota and colonic barrier gene correlations. **(A)** Alpha diversity (ACE, Chao1, Shannon, and Simpson indices). **(B)** Principal coordinate analysis (PCoA). **(C)** Nonmetric multidimensional scaling (NMDS) analysis. Composition of the gut microbiome (>1% relative abundance) at the **(D)** phylum, **(E)** family, and **(F)** genus levels. **(G)** Intergroup genus-level abundance differences. BD+NB, piglets received the basal diet + oral nutrient broth; LN+NB, piglets received the basal diet supplemented with 150 mg/kg linarin + oral nutrient broth; BD+ETEC, ETEC-challenged piglets fed the basal diet; LN+ETEC, piglets with ETEC infection fed the basal diet + 150 mg/kg linarin. Significance thresholds are set at *P* < 0.05 (statistically significant) and *P* < 0.01 (extremely statistically significant). *: *P* < 0.05 (statistically significant), **: *P* < 0.01 (highly statistically significant).

The ACE index for the LN+NB group was significantly (*P* < 0.05) reduced compared to that of the BD+NB group, whereas no notable alterations were observed in the observed species, Chao1, Shannon, and Simpson indices between these two groups (*P* > 0.05) (**Figure 4A**). Principal Coordinates Analysis (PCoA) and Non-metric Multidimensional Scaling (NMDS) analysis indicated significant differences in the structural features of the gut microbiota composition between the BD+NB and LN+NB groups (**Figures 4B, C**). Firmicutes and Bacteroidota were the dominant phyla, followed by Euryarchaeota, Proteobacteria, and Spirochaetota (**Figure 4D**). A total of 20 families (**Figure 4E**) and 26 genera (**Figure 4F**) had a major contribution to the observed differences, based on a relative abundance exceeding 1%. At the genus level, the relative abundances of *Sarcina*, *Roseimarinus*, *Fibrobacter*, and *Mesoplasma* were higher in the LN+NB group than in the BD+NB group (*P* < 0.05), whereas *Tyzzerella*, *Ralstonia*, *Anaerofilum*, *Bose* (*P* < 0.01), *Catenibacillus*, and *Faecalibacterium* decreased in abundance with linarin supplementation (*P* < 0.05) (**Figure 4G**).

**Figure 5:**
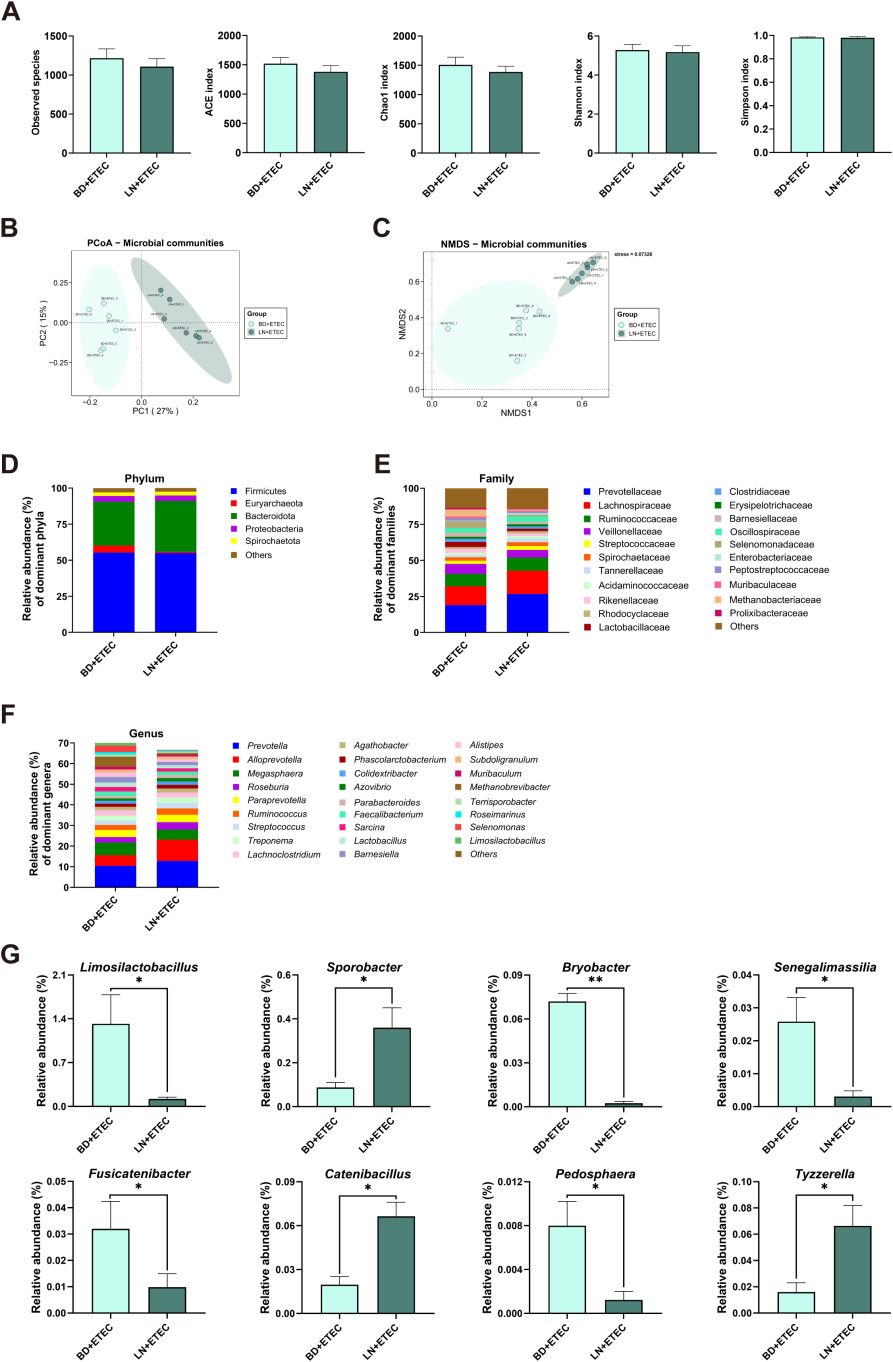
Effects of dietary linarin on weaned piglet microbiota and colon barrier gene correlations after ETEC infection. **(A)** Alpha diversity (ACE, Chao1, Shannon, and Simpson indices). **(B)** Principal coordinate analysis (PCoA). **(C)** Nonmetric multidimensional scaling (NMDS) analysis. Composition of the gut microbiome (>1% relative abundance) at the **(D)** phylum, **(E)** family, and **(F)** genus levels. **(G)** Intergroup genus-level abundance differences. BD+NB, piglets received the basal diet + oral nutrient broth; LN+NB, piglets received the basal diet supplemented with 150 mg/kg linarin + oral nutrient broth; BD+ETEC, ETEC-challenged piglets fed the basal diet; LN+ETEC, piglets with ETEC infection fed the basal diet + 150 mg/kg linarin. Significance thresholds are set at *P* < 0.05 (statistically significant) and *P* < 0.01 (extremely statistically significant). *: *P* < 0.05 (statistically significant), **: *P* < 0.01 (highly statistically significant).

Linarin supplementation did not have an influence on the microbiota diversity in the context of ETEC infection, with no significant differences in any of the diversity indices (observed species, Chao1, ACE, Shannon, Simpson) noted between the LN+ETEC and BD+ETEC groups (all *P* > 0.05) (**Figure 5A**). Nevertheless, PCoA and NMDS analysis revealed distinct gut microbiota structures between the BD+ETEC and LN+ETEC groups (**Figures 5B, C**). Firmicutes and Bacteroidota were the dominant phyla, followed by Euryarchaeota, Proteobacteria, and Spirochaetota (**Figure 5D**). A total of 21 families (**Figure 5E**) and 26 genera (**Figure 5F**) had major contributions to the differences, with relative abundances exceeding 1%. At the genus level, the abundances of *Sporobacter*, *Catenibacillus*, and *Tyzzerella* were significantly (*P* < 0.05) higher, whereas the abundances of *Limonolobacillus*, *Bryobacter*, *Senecamassilia*, *Fusicatenibacter*, and *Pedosphaera* were significantly (*P* < 0.05) lower in the LN+ETEC group compared to the BD+ETEC group (**Figure 5G**).

### 3.10 Non-targeted metabolomic analysis

**Figure 6:**
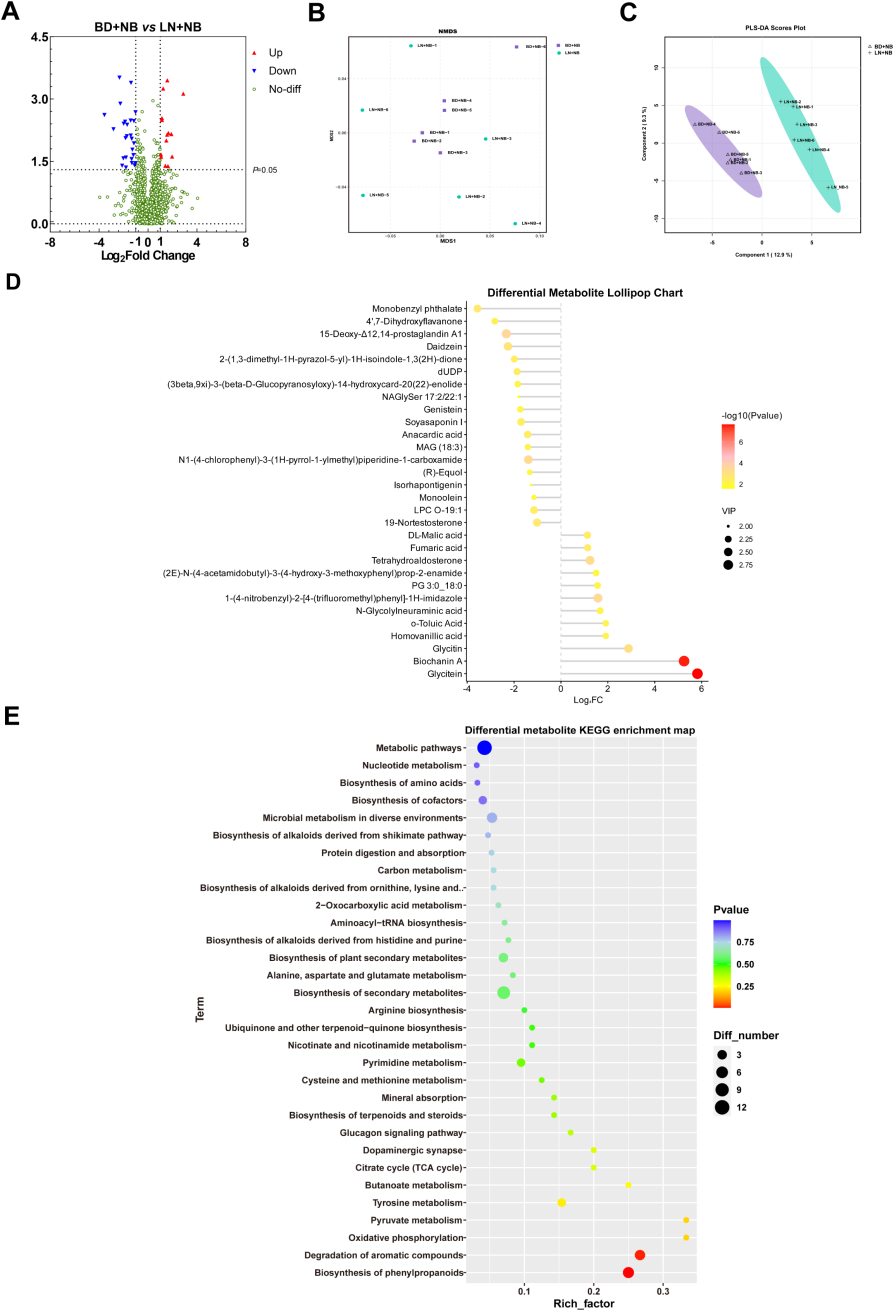
Effects of dietary linarin on the metabolites in the colonic chyme of weaned piglets. **(A)** Volcano plots indicating significant metabolites [log2 fold change (FC) ≥ 1; *P* < 0.05]. **(B)** Non-metric multidimensional scaling (NMDS) analysis. **(C)** Partial least-squares discriminant analysis (PLS-DA). **(D)** Differential metabolite lollipop chart. **(E)** Differential metabolite KEGG enrichment map.

As shown in the volcano diagram in **Figure 6A**, we found 1709 differentially expressed metabolites between the BD+NB and LN+NB groups, including 17 upregulated metabolites and 26 downregulated metabolites, following linarin supplementation. Based on Bray–Curtis distances, the NMDS analysis and PLS-DA showed that the microbial community structures of the BD+NB and LN+NB groups differed substantially (**Figures 6B, C**). The lollipop chart in **Figure 6D** shows that 12 of the differentially expressed metabolites, including glycitin and lycotein, were significantly upregulated, whereas 18 of the differentially expressed metabolites, including daidzein, genistein, (R)-equol, and anacardic acid, were significantly downregulated with linarin supplementation. KEGG enrichment analysis demonstrated that dietary linarin significantly modulated 31 key metabolic pathways, including biosynthesis of cofactors, oxidative phosphorylation, carbon metabolism, and methionine metabolism (**Figure 6E**).

**Figure 7:**
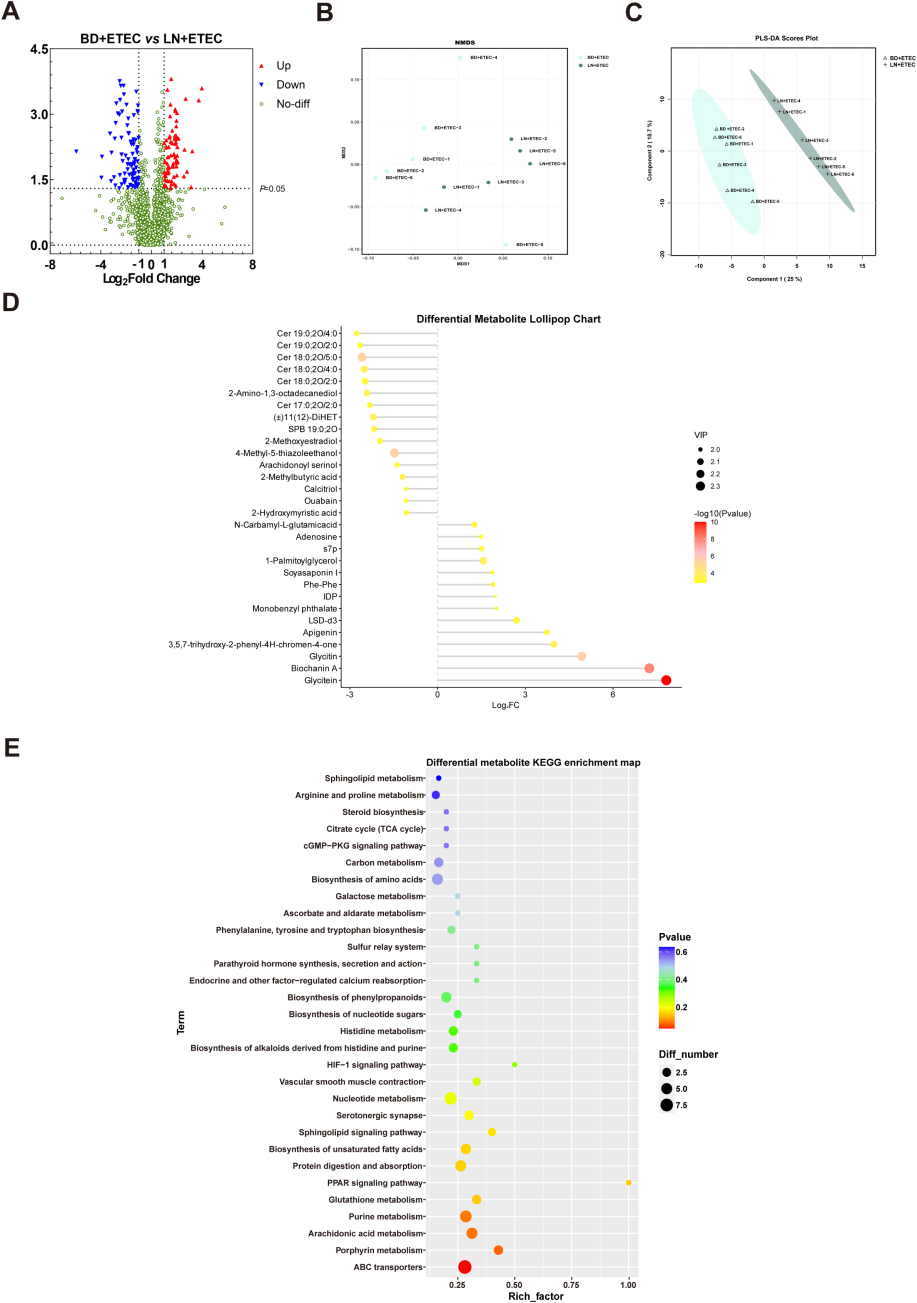
Effects of linarin on the metabolic products of the colonic chyme. **(A)** Volcano plots indicating significant metabolites [log2 fold change (FC) ≥ 1; *P* < 0.05]. **(B)** Non-metric multidimensional scaling (NMDS) analysis. **(C)** Partial least-squares discriminant analysis (PLS-DA). **(D)** Differential metabolite lollipop chart. **(E)** Differential metabolite KEGG enrichment map.

The volcano diagram in **Figure 7A** shows the 1709 differentially expressed metabolites between the BD+ETEC and LN+ETEC groups, including 82 upregulated metabolites and 90 downregulated metabolites. According to Bray–Curtis distances, the NMDS analysis and PLS-DA identified significant variations in microbial community composition between these two groups (**Figures 7B, C**). The lollipop chart in **Figure 7D** demonstrates that 14 differentially expressed metabolites, including biochanin A, monobenzyl phthalate, and glycitein, showed significant upregulation, whereas 12 differentially expressed metabolites, including calcitriol, 2-methoxyestradiol, and ouabain, showed significant downregulation after linarin supplementation in the context of ETEC infection. KEGG enrichment analysis showed that linarin with ETEC infection significantly modulated 30 key metabolic pathways, including the glutathione and sphingolipid metabolism pathways (**Figure 7E**).

### 3.11 Correlation analysis

#### 3.11.1 Correlations among the gut microbiome, intestinal barrier function, apoptosis, and TLR4 signaling pathway

**Figure 8:**
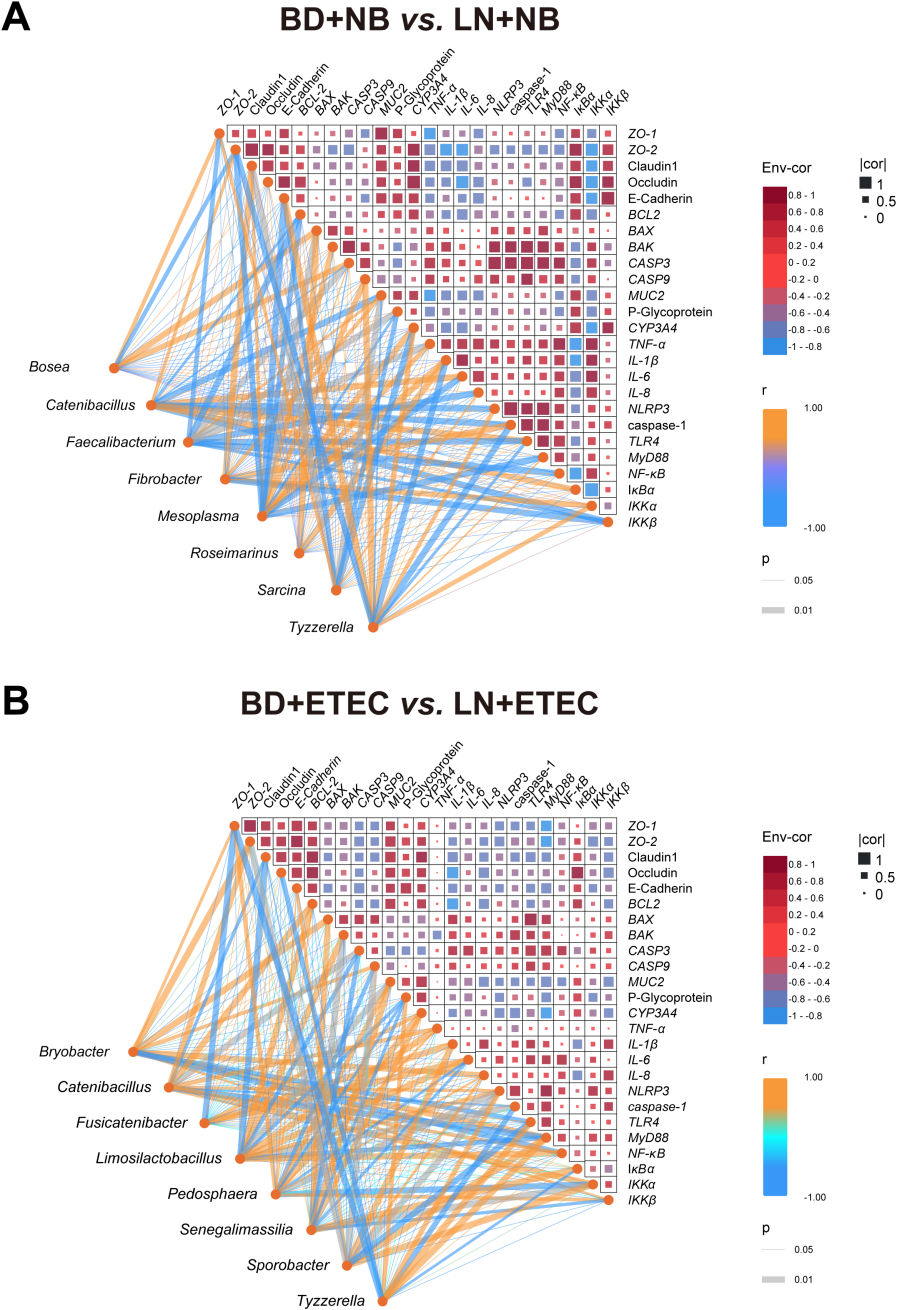
Correlations between microbiota abundance at the genus level and intestinal barrier/apoptosis/TLR4 signaling pathway-related gene expression in weaned piglets. **(A)** BD+NB *vs*. LN+NB comparison. **(B)** BD+ETEC *vs*. LN+ETEC comparison. BD+NB, piglets received the basal diet + oral nutrient broth; LN+NB, piglets received the basal diet supplemented with 150 mg/kg linarin + oral nutrient broth; BD+ETEC, ETEC-challenged piglets fed the basal diet; LN+ETEC, piglets with ETEC infection fed the basal diet + 150 mg/kg linarin. Significance thresholds are set at *P* < 0.05 (statistically significant) and *P* < 0.01 (extremely statistically significant).

Correlation analysis was performed to explore the interactions among dietary linarin-induced alterations in the gut microbiota, colonic barrier function, apoptosis, and TLR4 signaling pathway in weaned piglets. *Bosea* abundance was positively correlated with *ZO-1* and *BAX* mRNA expression, whereas it was negatively correlated with *ZO-2* expression (*P* < 0.05). The relative abundance of *Catenibacillus* was positively correlated with *ZO-2*, *IL-6*, *NF-κB*, and *IκBα* expression levels, whereas it was negatively correlated with *BCL2*, *CASP3*, *MUC2*, *NLRP3*, caspase-1, and *IKKβ* mRNA expression levels (*P* < 0.05). The relative abundance of *Faecalibacterium* was positively correlated with *MUC2* expression, but was negatively correlated with *ZO-1*, *BCL2*, *CASP3*, P-glycoprotein, *TNF-α*, and *IL-1β* mRNA expression levels (*P* < 0.05). The relative abundance of *Fibrobacter* was positively correlated with claudin-1, *MUC2*, caspase-1, and *IKKα* mRNA expression levels and was negatively correlated with *IKKβ* expression (*P* < 0.05). The relative abundance of *Mesoplasma* was positively correlated with claudin-1, *BAK*, *CASP3*, *CASP9*, *CYP3A4*, and *TNF-α* expression, whereas it was negatively correlated with E-cadherin, *BCL2*, *BAX*, *MUC2*, P-glycoprotein, *IL-6*, and *MyD88* mRNA expression levels (*P* < 0.05). *Roseimarinus* abundance was positively associated with *BAX*, *IL-1β*, and *TLR4* expression, but was negatively correlated with *CASP3* and *TNF-α* expression (*P* < 0.05). The relative abundance of *Sarcina* was positively correlated with *ZO-1*, *IL-8*, and *IκBα* levels, whereas it was negatively correlated with claudin-1, E-cadherin, *MUC2*, P-glycoprotein, *CYP3A4*, and *MyD88* mRNA expression levels (*P* < 0.05). The relative abundance of *Tyzzerella* was positively correlated with E-cadherin, *CASP9*, *CYP3A4*, *NLRP3*, *MyD88*, and *IKKα* expression and was negatively correlated with *MUC2*, *IL-1β*, caspase-1, and *TLR4* mRNA expression levels (*P* < 0.05) (**Figure 8A**).

Under ETEC infection with and without linarin supplementation, *Bryobacter* abundance showed a positive correlation with *ZO-1*, *ZO-2*, *BAX*, *CASP9*, *IL-8*, and *MyD88* expression, whereas it was negatively correlated with *TLR4* and *NF-κB* expression (*P* < 0.05). The relative abundance of *Catenibacillus* was positively correlated with *ZO-2*, *BCL2*, *TNF-α*, *NLRP3*, *TLR4*, *NF-κB*, and *IKKα* expression, but was negatively correlated with claudin-1, *CASP3*, caspase-1, and *MyD88* mRNA expression levels (*P* < 0.05). *Fusicatenibacter* abundance was positively correlated with *CYP3A4*, *IL-1β*, and *IL-6* expression, whereas it was negatively correlated with *ZO-1* expression (*P* < 0.05). The relative abundance of *Limosillactobacillus* was positively correlated with *BCL2*, *BAK*, *TNF-α*, *NLRP3*, and *NF-κB* expression, whereas it was negatively correlated with claudin-1, *CASP3*, *MUC2*, P-glycoprotein, caspase-1, and *MyD88* mRNA expression levels (*P* < 0.05). The relative abundance of *Pedosphaera* was positively correlated with *ZO-1*, E-cadherin, *BCL2*, *MUC2*, *CYP3A4*, *TNF-α*, *IL-8*, and *IKKβ* mRNA levels, but was negatively correlated with *CASP9*, P-glycoprotein, *IL-6*, *NLRP3*, and *IKKα* mRNA expression levels (*P* < 0.05). *Senegalimassilia* abundance was positively correlated with *BAX*, *MUC2*, *CYP3A4*, and *IκBα*, but was negatively correlated with *BAK*, *CASP3*, P-glycoprotein, *IL-6*, *IKKα*, and *IKKβ* mRNA expression (*P* < 0.05). The relative abundance of *Sporobacter* was positively correlated with E-cadherin, *IL-1β*, *IL-8*, *MyD88*, and *IKKα*, whereas it was negatively correlated with *ZO-2*, claudin-1, *BAK*, *CASP9*, *NLRP3*, and *TLR4* mRNA expression levels (*P* < 0.05). The relative abundance of *Tyzzerella* was positively correlated with P-glycoprotein, *NLRP3*, *TLR4*, and *MyD88*, but showed an inverse relationship with *ZO-1*, occludin, caspase-1, and *IκBα* mRNA expression levels (*P* < 0.05) (**Figure 8B**).

#### 3.11.2 Correlations among microorganisms and metabolic pathways

**Figure 9:**
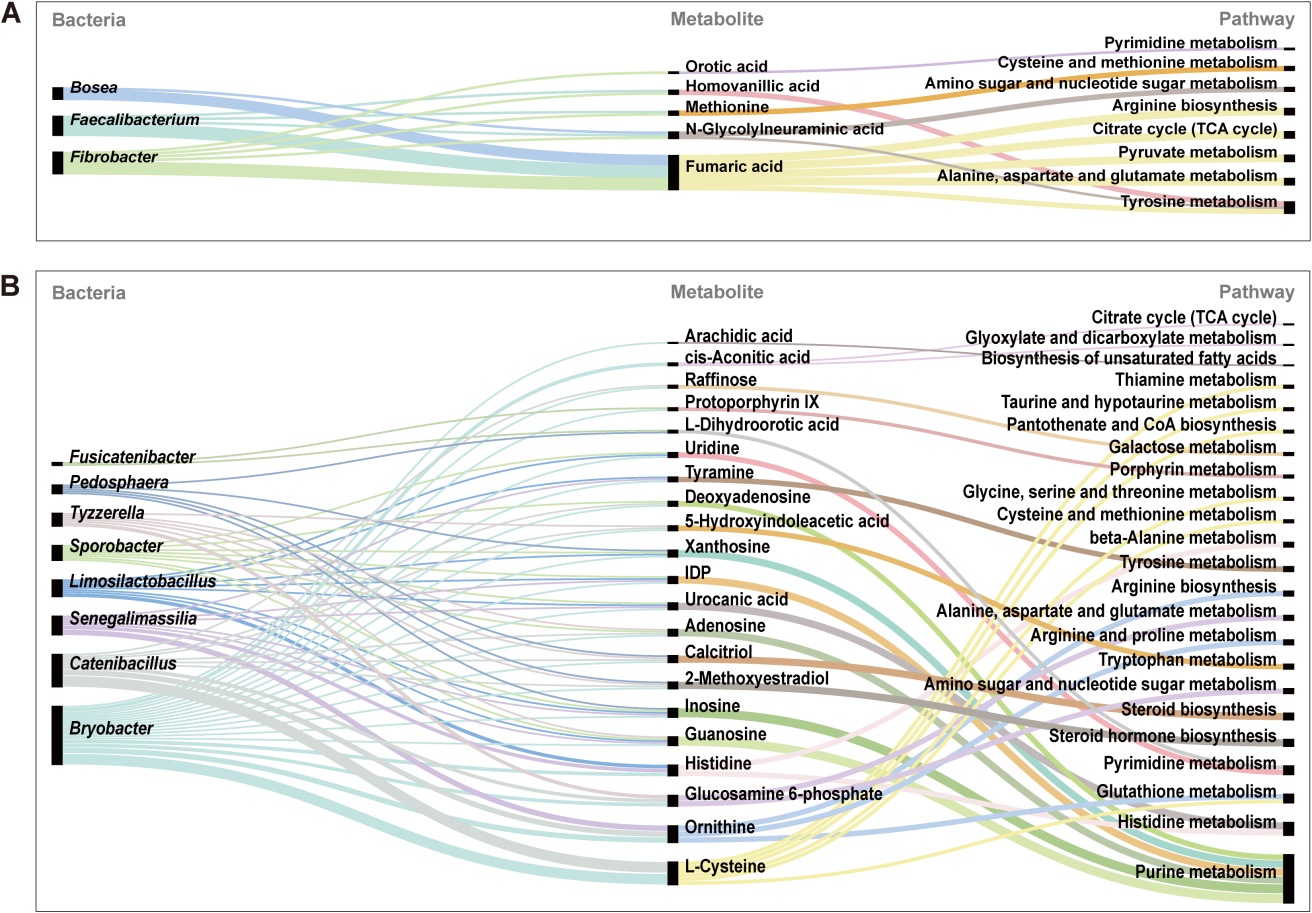
Relationships among colonic microbiota, metabolites, and metabolic pathways. **(A)** BD+NB *vs*. LN+NB comparison. **(B)** BD+ETEC *vs*. LN+ETEC comparison. BD+NB, piglets received the basal diet + oral nutrient broth; LN+NB, piglets received the basal diet supplemented with 150 mg/kg linarin + oral nutrient broth; BD+ETEC, ETEC-challenged piglets fed the basal diet; LN+ETEC, piglets with ETEC infection fed the basal diet + 150 mg/kg linarin. Significance thresholds are set at *P* < 0.05 (statistically significant) and *P* < 0.01 (extremely statistically significant).

Correlation analysis was further conducted to examine the associations of the dietary linarin-induced alterations in gut microbiota with differential metabolites and metabolic pathways in weaned piglets. The relative abundance of *Faecalibacterium* was positively correlated with tyrosine metabolism, arginine biosynthesis, citrate cycle [i.e., the tricarboxylic acid (TCA) cycle], pyruvate metabolism, alanine/aspartate/glutamate metabolism, amino/nucleotide sugar metabolism, and cysteine/methionine metabolism. The relative abundance of *Bosea* was negatively correlated with arginine biosynthesis; pyruvate metabolism; alanine, aspartate, and glutamate metabolism; and tyrosine metabolism. *Fibrobacter* abundance showed a positive link to tyrosine metabolism; arginine biosynthesis; TCA cycle; pyruvate metabolism; alanine, aspartate, and glutamate metabolism; cysteine and methionine metabolism; and pyrimidine metabolism (**Figure 9A**).

Under ETEC infection with and without linarin supplementation, *Pedosphaera* abundance showed a positive link with purine and pyrimidine metabolism, but was negatively correlated with steroid biosynthesis and steroid hormone biosynthesis. The relative abundance of *Fusicatenibacter* was positively correlated with porphyrin metabolism and pyrimidine metabolism. The relative abundance of *Tyzzerella* was positively correlated with alanine, aspartate, and glutamate metabolism; steroid biosynthesis; tryptophan metabolism; amino sugar and nucleotide sugar metabolism; and steroid hormone biosynthesis. *Sporobacter* abundance was positively correlated with purine, histidine, and pyrimidine metabolism. *Limosilactobacillus* abundance showed a positive correlation with purine, histidine, β-alanine, and pyrimidine metabolism, but was negatively correlated with tyrosine metabolism. The abundance of *Senegalimassilia* showed a positive association with arginine biosynthesis, glutathione metabolism, and tyrosine and proline metabolism, whereas it was negatively correlated with histidine, purine, and β-alanine metabolism. *Catenibacillus* abundance was positively linked to pathways involving purine and amino acid metabolism, steroid biosynthesis, tryptophan, and sugar metabolism, whereas negative correlations were found with glutathione, thiamine, taurine, pantothenate, galactose, glycine/serine/threonine, cysteine/methionine, and steroid hormone metabolism. *Bryobacter* abundance showed positive correlations to glutathione, arginine, and pantothenate/CoA biosynthesis; porphyrin, glycine, serine, threonine, cysteine, methionine, and fatty acids metabolism; and steroid and steroid hormone biosynthesis. However, the relative abundance of *Bryobacter* was negatively correlated with thiamine, taurine, and hypotaurine metabolism; the TCA cycle; β-alanine metabolism; galactose metabolism; alanine, aspartate, and glutamate metabolism; glyoxylate and dicarboxylate metabolism; pyrimidine metabolism; and tryptophan metabolism (**Figure 9B**).

## 4 Discussion

Weaning under stress-induced intestinal barrier dysfunction represents a critical constraint on piglet health and growth. This vulnerability is further compounded by ETEC infection, which exacerbates intestinal permeability, disrupts tight junction-proteins, and triggers inflammatory cascades, ultimately resulting in diarrhea and growth retardation (23, 24). Owing to their anti-inflammatory, antioxidant, and gut-modulating activities, plant-derived bioactive compounds have emerged as viable antibiotic substitutes in feed formulations. Among these, linarin, a flavonoid that is abundant in Asteraceae species, has demonstrated the ability to enhance intestinal immunity and hinder pathogen adhesion (10). In this study, combined analysis of intestinal morphology, tight-junction mRNA expression profiles, and inflammatory signaling pathways in a piglet model infected with ETEC provides the first demonstration that linarin can alleviate intestinal barrier damage through a multi-target regulatory mechanism.

ETEC invasion may disturb the balance of gut microbiota, thereby inducing diarrhea (4). The broad biological activities of flavonoids are well established, including antibacterial and inflammation-reducing effects, along with their ability to regulate the structure and composition of gut microbiota, which can help alleviate diarrhea. In line with this previous work, the present study indicated that dietary linarin significantly decreased the diarrhea rate of piglets, even in the context of ETEC infection.

Consistent with reports that ETEC challenge reduces colonic crypt depth (25), we observed similar morphological damage. Crucially, dietary linarin significantly increased crypt depth, suggesting it counteracts damage caused by ETEC and potentially enhances intestinal barrier integrity. Furthermore, our finding that linarin markedly boosted goblet cell counts under both basal conditions and following ETEC challenge aligns with the established role of these cells in mucosal protection and barrier function, and their depletion during ETEC pathogenesis (26). These findings suggest that linarin may promote intestinal repair and improve mucosal protection by supporting goblet cell function, thereby strengthening the intestinal barrier.

We further investigated the impact of dietary linarin on the physical barrier of the colon. In this study, dietary linarin significantly increased the mRNA expression levels of *ZO-1*, *ZO-2*, claudin-1, occludin, and E-cadherin, both under basal conditions and following ETEC challenge in weaned piglets. This suggests that linarin may attenuate the ETEC-induced disruption of colonic tight and adhesive junctions. Mechanistically, linarin’s barrier-fortifying effect aligns with the established role of flavonoids like quercetin and rutin, which enhance intestinal barrier function by promoting junctional protein expression during stress or infection (13), thereby improving intestinal barrier integrity and potentially mitigating ETEC-induced injury.

Intestinal pathogens such as ETEC exploit host cell apoptosis to enhance their survival and dissemination during infection (27). In this study, dietary linarin significantly upregulated the mRNA expression level of *BCL2* while reducing the levels of *BAX*, *BAK*, *CASP3*, and *CASP9* in weaned piglets. This suggests that linarin may alleviate ETEC-induced apoptosis by enhancing anti-apoptotic signals and inhibiting pro-apoptotic pathways, thereby protecting intestinal cells from pathogen-induced damage.

The intestinal chemical barrier, comprising secreted antimicrobial factors, is critical for defense against pathogens like ETEC (16). In this study, dietary linarin significantly increased *MUC2*, *CYP3A4*, and P-glycoprotein mRNA expression levels. This suggests that linarin could enhance intestinal chemical barrier function by upregulating key defense markers (*MUC2*, *CYP3A4*, and P-glycoprotein) in weaned piglets after ETEC infection.

The intestinal immune barrier regulates gut microbiota homeostasis by suppressing pathogens while preserving commensal bacteria (28). In this study, dietary linarin significantly decreased the mRNA expression levels of *TNF-α*, *IL-1β*, *IL-6*, and *IL-8* in both healthy and ETEC-challenged weaned piglets This downregulation suggests that linarin helps maintain immune barrier integrity during ETEC infection by limiting cytokine-driven epithelial damage and inflammatory cell infiltration, thereby preserving gut homeostasis and improving resistance to enteric pathogens.

To further elucidate the mechanisms by which linarin mitigates ETEC-induced inflammation, we examined its influence on the TLR4/MyD88/NF-κB signaling pathway. ETEC-derived lipopolysaccharide binds to TLR4 on epithelial cells, initiating downstream MyD88 recruitment and NF-κB activation. This process activates NLRP3 inflammasomes and pro-inflammatory cytokines through caspase-1 activation. *IκBα*, a key *NF-κB* inhibitor, is phosphorylated and degraded upon IKK complex activation, allowing *NF-κB* nuclear translocation and cytokine transcription; IKKα and IKKβ subunits are central to this process (29, 30). In this study, dietary linarin significantly decreased the mRNA expression levels of *TLR4*, *MyD88*, *NF-κB*, *NLRP3*, *IKKα*, and caspase-1, both without and with ETEC infection. Overall, these findings indicate that dietary linarin could suppress the NF-κB/MyD88/TLR4 signaling pathway and downstream IKK complex activation in intestinal epithelial cells, block activation of the NLRP3 inflammasome and caspase-1, and significantly reduce the mRNA expression of *TNF-α*, *IL-1β*, *IL-6*, and *IL-8*, ultimately enhancing gut immune defenses and mitigating ETEC-induced inflammation.

Short-chain fatty acids (SCFAs) are metabolites derived from the fermentation of dietary fiber by commensal gut bacteria, which modulate gut health and immune cell function (31). Among the main SCFAs, butyrate is essential for immune modulation and barrier function (32); acetic acid regulates immune responses and energy metabolism by activating G protein-coupled receptors, thereby modulating immune cell function and inhibiting inflammation; and propionic acid exerts anti-inflammatory and immunomodulatory effects, reducing pro-inflammatory cytokines in inflammatory bowel disease (33). A previous study showed that ETEC infection reduces SCFAs in piglet intestines (34). In this study, dietary linarin (LN+NB) significantly elevated the concentrations of acetic, propionic, butyric, and isovaleric acids. Similarly, after ETEC infection, dietary linarin significantly increased acetic, propionic, butyric, valeric, and isovaleric acid levels. This suggests that linarin could elevate intestinal barrier function and adjust the immune response by facilitating the synthesis of SCFAs.

The gut microbiome maintains intestinal barrier function by competitively inhibiting pathogens and producing antibacterial substances. In our study, linarin supplementation significantly reduced the relative abundances of several taxa—including *Faecalibacterium*, *Catenibacillus*, *Tyzzerella*, *Ralstonia*, *Bosea* and *Anaerofilum*—that are either associated with pro−inflammatory signaling or opportunistic pathogenicity (35–39). This shift may underlie the observed inhibition of the TLR4/NF−κB pathway. In contrast, linarin promoted the growth of strict anaerobes and SCFA producers such as *Sarcina*, *Roseimarinus*, *Fibrobacter* and *Mesoplasma* (40–43). By lowering colonic pH through butyrate and other SCFAs, and by reducing oxidative stress via its antioxidant properties, linarin created an environment favorable to beneficial anaerobes while suppressing taxa that exacerbate inflammation. Together, these data reveal that linarin confers protection against ETEC‐induced dysbiosis by both weakening a pro−inflammatory microbial milieu and fostering SCFA‐mediated barrier reinforcement.

After ETEC infection, dietary linarin significantly decreased the abundance of *Limosilobacillus*, *Bryobacter*, *Senecamassilia*, *Fusicatenibacter*, and *Pedosphaera*—genera involved in immunomodulation, carbon metabolism and mucosal colonization (44–48). This reduction may result from linarin’s antioxidant effect lowering intestinal ROS levels, potentially inhibiting redox-signaling reliant bacteria like *Bryobacter* and *Senecamassilia*, and its upregulation of tight-junction proteins, which reduces intestinal permeability and may restrict mucosal injury-dependent colonization by genera like *Senecamassilia*. Conversely, dietary linarin significantly increased the abundance of *Sporobacter*, *Catenibacillus*, and *Tyzzerella* (49), likely through enhanced substrate availability and niche exclusion of opportunists. Collectively, these shifts suggest linarin’s anti-inflammatory and antioxidant effects help alleviate ETEC-induced intestinal damage, creating conditions favorable for the proliferation of beneficial bacteria like *Sporobacter*, *Catenibacterium*, and *Tyzzerella*.

Metabolomics integrates high-throughput analysis and bioinformatics to assess metabolic responses. Our metabolomics analysis demonstrates that dietary linarin profoundly reshapes colonic metabolite networks by enhancing nucleotide metabolism, TCA cycle activity, and butyrate pathways. Upregulated nucleotide turnover may curb uric acid accumulation by suppressing uricogenic bacteria such as *Catenibacillus*, while increased TCA cycle flux supplies ATP to meet and thereby downregulate NF−κB–driven inflammatory demands (50). Concurrently, boosted butyrate metabolism not only generates anti−inflammatory SCFAs but also promotes a microbiota environment favorable to butyrogenic taxa. This suggests that linarin may reduce the production of proinflammatory metabolites, create a more favorable growth environment for butyric acid-producing bacteria, and indirectly promote butyric acid metabolism.

After ETEC infection, dietary linarin significantly affected the characteristic distribution of metabolites in the colonic chyme of weaned piglets. Linarin also rebalanced colonic metabolite profiles by modulating sphingolipid, carbon, and arginine–proline pathways. Sphingolipid metabolism attenuation restored enzymatic/metabolite equilibrium to suppress NF-κB-driven inflammation (51), while concurrent stabilization of carbon metabolism countered ETEC toxin-induced disruption of glycolysis and TCA cycling, thereby preserving host glucose homeostasis (52). Furthermore, linarin optimized arginine-proline metabolism to enhance proline hydroxylase activity and collagen synthesis, accelerating antioxidant-dependent mucosal repair. Collectively, these shifts illustrate how linarin orchestrates a metabolomic milieu conducive to energy homeostasis, redox balance, and inflammation resolution.

To further explore the impact of linarin on the gut microbiome, we evaluated the relationships among the key alterations in the microbiome with the changes in the expression of key marker genes associated with colonic barrier function, apoptosis, and the TLR4/MyD88/NF-κB signaling pathway. The result shows that dietary linarin intervention significantly changed the abundance of *Bosea*, *Catenibacillus*, *Faecalibacterium*, and other flora, which was in turn significantly related to the expression of genes associated with the colonic physical barrier, chemical barrier, immune barrier, apoptosis regulation, and the TLR4/MyD88/NF-κB signaling pathway. Therefore, linarin may synergistically enhance intestinal barrier function, maintain apoptosis balance, and inhibit an excessive inflammatory reaction by regulating the flora host gene interaction network.

Similarly, after ETEC infection with dietary linarin supplementation, this study showed that after ETEC infection, dietary linarin intervention was closely related to the intestinal physical barrier, apoptosis regulation, and TLR4/MyD88/NF-κB signal pathway-related gene expression by significantly changing the abundance of *Bryobacter*, *Catenibacillus*, *Limosilactobacillus*, and other flora. Therefore, linarin might alleviate ETEC-induced intestinal injury by reshaping the flora–host interaction network, synergistically strengthening the integrity of the intestinal barrier, regulating cell apoptosis, and inhibiting the inflammatory cascade reaction.

By combining microbiome and metabolome analyses, we mapped how linarin reshapes microbial–metabolic interactions to support colonic barrier function. By suppressing *Bosea*, linarin redirects arginine toward mucosal repair via polyamine synthesis while reducing nitric oxide-mediated oxidative stress (53). Concurrent enrichment of *Fibrobacter* enhances fiber fermentation into short-chain fatty acids, reactivating the TCA cycle to fuel colonocyte energy demands (54). Reduction of *Faecalibacterium* preserves cysteine/methionine metabolism for sustained glutathione synthesis, maintaining antioxidant defenses (35). Post-ETEC infection, *Fusicatenibacter*-mediated porphyrin and pyrimidine metabolism further supports heme synthesis and epithelial regeneration (55). The link of this genus to pyrimidine metabolism further suggests a role in nucleotide synthesis for epithelial repair. These effects collectively mitigate ETEC-induced oxidative stress and maintain mucosal integrity.

After ETEC infection, linarin selectively suppressed genera tied to pro−inflammatory or dysregulated metabolism while enriching those supporting repair and homeostasis. By suppressing *Tyzzerella*, linarin attenuated corticoid-mediated inflammation and kynurenine pathway activation, while concurrently enriching Catenibacillus to convert linarin into bioactive quercetin, thereby inhibiting ETEC virulence and optimizing nitrogen metabolism for mucosal repair (56). Furthermore, linarin fine-tuned *Limosilactobacillus* to enhance carnosine synthesis and counter tyrosine-derived inflammatory mediators (57), and reduced *Senegalimassilia* to redirect arginine toward polyamine synthesis while mitigating histamine overproduction (46), Notably, *Bryobacter* downregulation balanced glutathione-dependent antioxidant defenses against pro-inflammatory steroid biosynthesis (58). Further illustrate how linarin orchestrates a metabolic shift toward energy provision, antioxidant defense, and reduced inflammation, thereby fostering mucosal repair.

Overall, these results show that dietary linarin maintained intestinal energy homeostasis and barrier function by changing the abundance of *Bosea*, *Faecalibacterium*, and *Fibrobacter*, controlling essential pathways in amino acids, the TCA cycle, and carbohydrate metabolism. After ETEC infection, linarin affected lipid metabolism, immune regulation, and nucleotide metabolism pathways by regulating the abundance of bacteria such as *Fusicatonibacter* and *Limosilactobacillus*, thereby alleviating inflammation and repairing the pathogen-induced intestinal damage.

## 5 Conclusion

This study demonstrated that dietary linarin significantly reduced the incidence of diarrhea in weaned piglets infected with ETEC by enhancing colonic barrier function at multiple levels. Specifically, linarin improved crypt architecture of colon, increased goblet cell numbers, upregulated the expression of key physical barrier genes, and inhibited the apoptosis of intestinal epithelial cells. At the signaling level, linarin exerted anti-inflammatory effects by regulating the TLR4/MyD88/NF-κB pathway. Moreover, linarin modulated the chemical barrier of the intestine by suppressing inflammatory cytokine overexpression and downregulating NLRP3 inflammasome and caspase-1 levels, thereby attenuating the inflammatory response. Notably, linarin elevated the concentrations of SCFAs in the colon, reshaped the microbiota structure, and influenced the interaction network between key bacterial taxa and metabolic pathways related to energy, amino acid, and nucleic acid metabolism.

To our knowledge, this is the first study to directly investigate the protective effects of linarin against ETEC infection in weaned piglets. These findings highlight the regulatory relationships between linarin supplementation and key mechanisms of infection—including colonic microbiota remodeling, intestinal barrier enhancement, apoptosis suppression, and signaling pathway modulation—alongside associated metabolite and metabolic pathway alterations. Future research will focus on characterizing the microbiota–metabolite–host cross-talk underlying linarin-mediated intestinal barrier repair. Collectively, this study offers novel mechanistic insights into how linarin mitigates ETEC-induced intestinal injury in weaned piglets, underscoring its potential as a safe alternative for improving gut health.

## Data Availability

The datasets presented in this study can be found in online repositories. The names of the repository/repositories and accession number(s) can be found in the article/**Supplementary Material**.

## Glossary

ACE: a microbial diversity index
BAK: BCL2-antagonist/killer 1
BAX: BCL2-associated X protein
BCL2: B-cell lymphoma 2
CASP3: caspase 3
CASP9: caspase 9
CYP3A4: cytochrome P450 3A4
DR: diarrhea rate
ERK: extracellular signal-regulated kinase
ETEC: enterotoxigenic Escherichia coli
H&E: hematoxylin & eosin
HPLC: high-performance liquid chromatography
IKKα: inhibitor of nuclear factor kappa-B kinase subunit alpha
IKKβ: inhibitor of nuclear factor kappa-B kinase subunit beta
IκBα: nuclear factor of kappa light polypeptide gene enhancer in B-cells inhibitor alpha
IL-1β: interleukin-1 beta
IL-6: interleukin-6
IL-8: interleukin-8
IRAK1: interleukin-1 receptor associated kinase 1
JNK: c-Jun N-terminal kinase
KEGG: kyoto encyclopedia of genes and genomes
MUC2: mucin 2
MyD88: myeloid differentiation primary response 88
NF-κB: nuclear factor kappa-B
NLRP3: nucleotide-binding oligomerization domain-like receptor family pyrin domain-containing 3
NMDS: non-metric multidimensional scaling
NO: nitric oxide
P-glycoprotein: permeability glycoprotein
PCoA: principal coordinate analysis
PCA/PLS-DA: principal component analysis/partial least squares discriminant analysis
PWD: post-weaning diarrhea
qPCR: real-time quantitative polymerase chain reaction
ROS: reactive oxygen species
SCFA: short-chain fatty acid
SEM: standard error of the mean
SPSS: statistical package for the social sciences
TCA cycle: tricarboxylic acid cycle
TLR4: toll-like receptor 4
TNF-α: tumor necrosis factor-alpha
TRAF6: TNF receptor associated factor 6
UHPLC-MS/MS: ultra-high performance liquid chromatography-tandem mass spectrometry.
