# Supplementary material for: Linarin alleviates colonic barrier dysfunction induced by enterotoxic Escherichia coli in weaned piglets by regulating the gut microbiota and metabolic pathways
Source: Front Immunol. 2025 Oct 23;16:1631991. doi: 10.3389/fimmu.2025.1631991 (PMC12588845; doi:10.3389/fimmu.2025.1631991)
Supplement: Supplementary file 1 [file DataSheet1.docx]

Supplementary Material

# Supplementary Figures and Tables

**Table S1**. Ingredients composition and nutrient specification of basal diet

| Items (%) | | Basal diet |
| --- | --- | --- |
| Corn | | 38.00 |
| Extruded corn | | 15.00 |
| Soybean meal | | 12.00 |
| Extruded soybean | | 8.00 |
| Fermented soybean meal | | 8.00 |
| Whey power | | 5.00 |
| Fish meal | | 2.00 |
| Flour | | 5.00 |
| Soybean oil | | 2.00 |
| Vitamin and mineral premix* | | 5.00 |
| Analyzed nutrient specification (%) |  | |
| Digestible energy/MJ·kg^-1^ | 14.23 | |
| Dry matter | | 88.4 |
| Crude ash | | 5.13 |
| Crude protein | | 18.56 |
| Ether extract | | 6.05 |
| Crude fiber | | 4.20 |
| Ca | | 0.59 |
| Total P | | 0.54 |

*The premix is mainly composed of minerals, vitamins, trace elements, and amino acids. Provided per kilogram of diet: vitamin A, 12000 IU; vitamin D3, 4800 IU; vitamin E, 80 IU; vitamin K3, 3.0 mg; vitamin B1, 8.5 mg; vitamin B2, 12.0 mg; vitamin B6, 8.0 mg; vitamin B12, 0.08 mg；biotin, 0.30 mg; folic acid, 1.2 mg; pantothenic acid, 50.0 mg; nicotinic acid, 75 mg; Cu (CuSO_4_·5H_2_O), 80 mg; Fe (FeSO_4_·H_2_O), 135 mg; Zn (ZnSO_4_·H_2_O), 1500 mg; Mn (MnSO_4_·H_2_O), 30 mg; Se (Na_2_SeO_3_), 0.30 mg; I (Ca(IO_3_)_2_, 0.6 mg.

**Table S2** Sequences of genes-special primers used for RT-qPCR.

| Genes | Accession number | Primer sequences (5’-3’) | Amplicon size | Annealing temperature |
| --- | --- | --- | --- | --- |
| *ZO-1* | XM_021098896.1 | F: CCTGAGTTTGATAGTGGCGTTGA  R: AAATAGATTTCCTGCTCAATTCC | 269 | 60.9 |
| *ZO-2* | NM_001206404.1 | F: GCAGAGACAACCCCCACTTT  R: CGTTAACCATGACCACCCGA | 117 | 60.2 |
| Claudin1 | NM_001244539.1 | F: AAGATGCGGATGGCTGTCATT  R: ATTGACTGGGGTCATGGGGTC | 126 | 60.4 |
| Occludin | XM_005672525.3 | F: ACCCAGCAACGACATA  R: TCACGATAACGAGCATA | 155 | 51.9 |
| E-cadherin | NM_001163060.1 | F: AATGATGTGGCACCAACCCT  R: TAGCAGCTTCGGAACCACTG | 178 | 59.9 |
| *BCL2* | XM_005666256.3 | F: AATGATGTGGCACCAACCCT  R: TAGCAGCTTCGGAACCACTG | 178 | 59.9 |
| *BAX* | XM_013998624.2 | F: GCTGACGGCAACTTCAACTG  R: GAAGGAAGTCCAGCGTCCAG | 133 | 60.0 |
| *BAK* | XM_021098603.1 | F: ATGACATCAACCGGCGATAC  R: TTGATGCCACTCTCGAACAG | 143 | 58.1 |
| *CASP3* | NM_214131.1 | F: TGGGATTGAGACGGACAGTG  R: CGCTGCACAAAGTGACTGGA | 157 | 59.4 |
| *MUC2* | NM_002457.5 | F: CACTACCCACCTGGAGCATC  R: GACGACTTGGGAGGAGTTGG | 78 | 59.8 |
| P-glycoprotein | NM_001308246.1 | F: GCCCAGATAACAGCACCACA  R: ATCTCCTGCCGCATGATAGC | 181 | 60.3 |
| *CYP3A4* | NM_001195509.1 | F: CCAAGGGGACCGTGATGATG  R: ATGCAGTTGCGGGGTCCAGT | 163 | 60.5 |
| *IL-1β* | NM_214055.1 | F: CCAGCCAGTCTTCATTGTTCAG  R: GCTGGATGCTCCCATTTCTC | 188 | 59.5 |
| *IL-6* | NM_214399.1 | F: TCCTCGGCAAAATCTCTGCAA  R: ACAAGACCGGTGGTGATTCTC | 181 | 60.3 |
| *IL-8* | NM_213867.1 | F: GCCTTCTTGGCAGTTTTCCTG  R: TGGAAAGGTGTGGAATGCGTA | 113 | 60.0 |
| *NLRP3* | NM_001256770.2 | F: TCTTTGGCTGTTCCTGAGGC  R: CTAGTCAGAGTCCCAGGGCA | 121 | 60.3 |
| *caspase-1* | NM_214162.1 | F: TACAAGAATCCCAGGCGGTG  R: CCTTTGGGCTATGTCTGGGG | 128 | 59.8 |
| *MyD88* | NM_001099923.1 | F: TGGAACAGACCAACTATCGGC  R: CATCAGAGACAACCACTACCATCC | 132 | 60.0 |
| *NF-κB* | NM_001114281.1 | F: CGGGGACTACGACCTGAATG  R: CGGGGACTACGACCTGAATG | 196 | 59.9 |
| *IκBα* | NM_020529.3 | F: GAAGTGATCCGCCAGGTGAA  R: CTGCTCACAGGCAAGGTGTA | 189 | 60.0 |
| *IKKα* | NM_001114279.1 | F: TTTTGGATGGAGTCAGGGGC  R: GTGTACTGCTTCGGCCCATA | 182 | 60.0 |
| *IKKβ* | XM_021077171.1 | F: GTGACATCGCCTCTGCACTTA  R: CTTGGCATACCCGAGGTCAA | 134 | 60.4 |
| *β-actin* | XM_021086047.1 | F: GCAAATGCTTCTAGGCGGAC  R: GCAAATGCTTCTAGGCGGAC | 148 | 59.6 |
| *GAPDH* | NM_001206359.1 | F: CCCTGCGCTCTCTGCTC  R: GCCAGAGTTAAAAGCAGCCC | 149 | 59.9 |
